# TLR2 Signaling Decreases Transmission of *Streptococcus pneumoniae* by Limiting Bacterial Shedding in an Infant Mouse Influenza A Co-infection Model

**DOI:** 10.1371/journal.ppat.1004339

**Published:** 2014-08-28

**Authors:** Aimee L. Richard, Steven J. Siegel, Jan Erikson, Jeffrey N. Weiser

**Affiliations:** 1 Department of Microbiology, University of Pennsylvania, Philadelphia, Pennsylvania, United States of America; 2 The Wistar Institute, Philadelphia, Pennsylvania, United States of America; The University of Texas Health Science Center at San Antonio, United States of America

## Abstract

While the importance of transmission of pathogens is widely accepted, there is currently little mechanistic understanding of this process. Nasal carriage of *Streptococcus pneumoniae* (the pneumococcus) is common in humans, especially in early childhood, and is a prerequisite for the development of disease and transmission among hosts. In this study, we adapted an infant mouse model to elucidate host determinants of transmission of *S. pneumoniae* from inoculated index mice to uninfected contact mice. In the context of co-infection with influenza A virus, the pneumococcus was transmitted among wildtype littermates, with approximately half of the contact mice acquiring colonization. Mice deficient for TLR2 were colonized to a similar density but transmitted *S. pneumoniae* more efficiently (100% transmission) than wildtype animals and showed decreased expression of interferon α and higher viral titers. The greater viral burden in *tlr2^−/−^* mice correlated with heightened inflammation, and was responsible for an increase in bacterial shedding from the mouse nose. The role of TLR2 signaling was confirmed by intranasal treatment of wildtype mice with the agonist Pam3Cys, which decreased inflammation and reduced bacterial shedding and transmission. Taken together, these results suggest that the innate immune response to influenza virus promotes bacterial shedding, allowing the bacteria to transit from host to host. These findings provide insight into the role of host factors in the increased pneumococcal carriage rates seen during flu season and contribute to our overall understanding of pathogen transmission.

## Author Summary

In this study, we sought to identify factors contributing to the transmission of the bacterial pathogen *Streptococcus pneumoniae* (the pneumococcus), a major cause of otitis media, pneumonia, and septicemia. Often found as a co-infection with other bacterial and viral pathogens, the pneumococcus is commonly carried by young children and is spread by close human contact, most likely through large droplet respiratory secretions. The specific determinants of bacterial transmission, however, have not been identified. This report details our use of an infant mouse model of transmission, which includes influenza A co-infection, to elucidate the mechanism of host-to-host transmission. We found that the inflammatory response to influenza, which is aggravated in the context of weakened host defense, promotes transmission by inducing bacterial shedding from the mouse nose. These results show how a bacterial pathogen exploits the host immune response to spread from one host to the next.

## Introduction

The bacterial pathogen *Streptococcus pneumoniae* (the pneumococcus) robustly colonizes the upper respiratory tract of humans and is commonly carried asymptomatically. Colonization rates are highest in early childhood, where they can exceed 80% [1]; in crowded environments such as daycare centers [2]; and when viral respiratory infections are prevalent [3]. From its niche in the nasopharynx, the bacterium can invade other host sites, and as a result the pneumococcus is a leading cause of otitis media, pneumonia, and septicemia. Importantly, colonization of the nasopharynx is the reservoir for pneumococcal disease [4]–[7] and transmission between hosts [8], [9]. While pneumococcal colonization and disease have been well-studied using animal models (for review see [10], [11]), transmission of the bacterium remains poorly understood. In humans, close contact is required for transmission, which is thought to occur via respiratory secretions, but the specific host and bacterial factors contributing to this process have not been elucidated [12], [13].

Recently, an infant mouse model of pneumococcal transmission has been described [14]. In this model, ‘index’ mice are given *Streptococcus pneumoniae* intranasally and co-housed with uninoculated ‘contact’ littermates. Subsequently, all pups are inoculated with influenza A virus and, following an exposure period, transmission from index to contact mice is assessed by enumerating bacteria in the nasopharynx. Using this model, this group demonstrated that both increasing the bacterial titer in the index mice and inducing inflammation in the contact mice led to more efficient transmission [15]. Given the timing of these experiments, these studies suggest a role for the innate immune response in the setting of co-infection in transmission of the pneumococcus from host to host.

The host immune response to influenza has been extensively reviewed [16], [17] and is briefly summarized here to highlight key elements. After passing through the mucus layer lining the respiratory tract, influenza A virus is recognized by several pattern recognition receptors (PRRs) expressed by epithelial cells, including the Toll-like receptors (TLRs), nucleotide oligomerization domain-like receptors (NLRs), and retinoic acid-inducible gene-I receptors (RIG-I). Engagement of these receptors induces a signaling cascade that culminates in expression of interferons (IFNs) and pro-inflammatory cytokines and chemokines, resulting in recruitment and activation of immune cells, such as neutrophils and macrophages. A recent study has shown that influenza infection also leads to increased mucin expression by epithelial cells in the respiratory tract [18].

TLR2 has been implicated in promoting clearance of the pneumococcus [19], [20], and stimulation of this receptor has been shown to protect against influenza infection [21]. Additionally, recent work has shown that signaling through TLR2 can induce anti-viral responses after stimulation by viral and synthetic ligands. [22], [23] Thus, we hypothesized that mice deficient for TLR2 would display altered transmission of the bacterium in this model. We found that mice deficient in TLR2 show heightened acute inflammation in index mice and enhanced transmission due to an increase in number of bacteria shed via nasal secretions. This model recapitulates many facets of human-to-human transmission of pneumococcal carriage, such as concurrent viral infection and close contact between infants/children, and these findings provide insight into how innate immune responses to infection promote the spread of pathogens from host to host.

## Results

### *S. pneumoniae* is transmitted between wildtype hosts in the context of influenza co-infection

**Figure 1:**
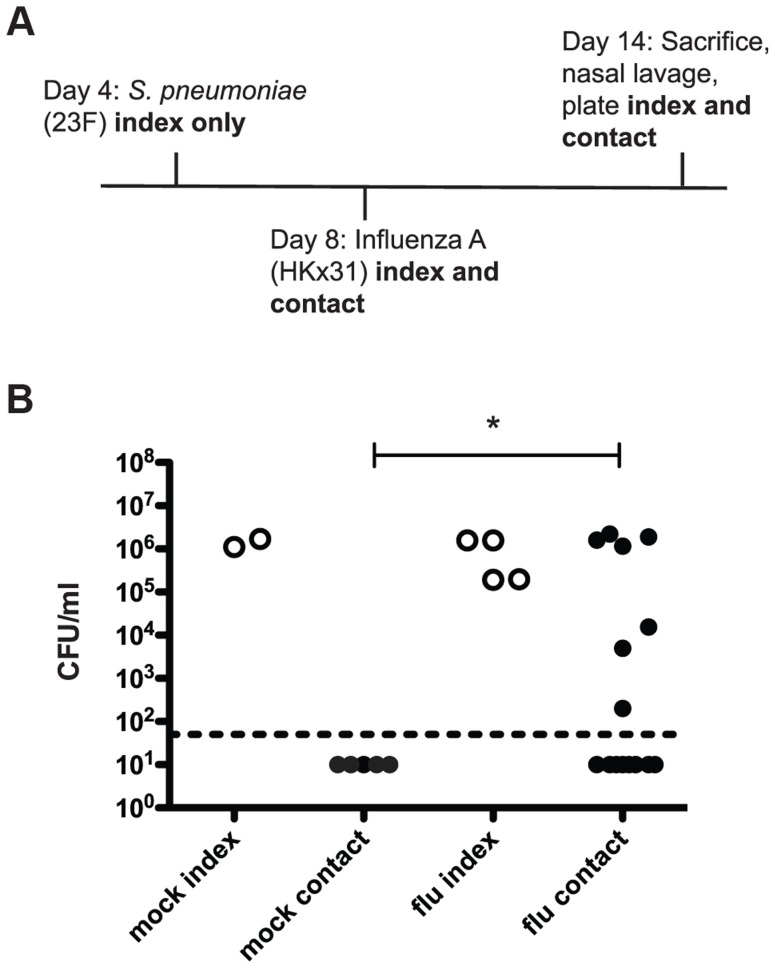
*S. pneumoniae* is transmitted between hosts in an infant mouse influenza co-infection model. **A.** Schematic of inoculation schedule. Listed agents were administered intranasally to unanesthetized mice in a volume of 3 µl PBS and pups were returned to their mothers. Numbered days refer to age of mice, which were euthanized on day 14. Nasal lavage was performed with 200 µl PBS, which was then serially diluted and plated on selective agar to obtain colony counts. **B.** Pups were infected and sacrificed as described above, with “mock” animals receiving 3 µl PBS on day 8 instead of influenza. Bacterial loads in nasal lavage fluid are shown. Each dot represents one mouse. Open circles designate index mice, and filled represent contact mice. Statistical significance was assessed using the Mann-Whitney test. Asterisk indicates *p*<0.05.

Our adaptations to the previously reported infant mouse model of pneumococcal transmission are outlined in the schematic in Figure 1A [14]. In these experiments, four-day-old pups were intranasally inoculated with pneumococci (index mice only) and on day 8 all mice in the litter (both index and contacts) were intranasally inoculated with Influenza A/HKx31 (mouse-adapted H3N2) virus. On day 14, the pups were sacrificed and bacterial loads were enumerated in nasal lavages. In wildtype mice, acquisition of colonization was detected in approximately half (47%) of the contact pups (Figure 1B, flu). In contrast, without influenza (PBS administered at day 8), none of the contact pups acquired *S. pneumoniae* (Figure 1B, mock). Moreover, transmission was not simply due to increased bacterial load in the index mice, as there was no significant difference between the mock or flu index groups. This phenotype makes this model particularly useful for probing the factors that limit and promote bacterial transmission because transmission can be either increased or decreased by experimental manipulations.

As this infection model utilizes a six-day exposure period, it is possible that contact mice infected early in that window could then go on to spread the bacteria to other contacts. In order to test this possibility, we repeated the initial experiment but gave *S. pneumoniae* to the index (“inoculated contact”) on day 9 instead of day 4, to simulate acquisition from an index mouse. These inoculated contacts were able to spread infection effectively, with four out of five “uninoculated contact” mice acquiring colonization (Figure S1). The observed transmission among contact mice makes it unlikely that the ratio of index to contact mice was a significant factor in overall rates of transmission.

### Mice lacking TLR2 display increased pneumococcal transmission

**Figure 2:**
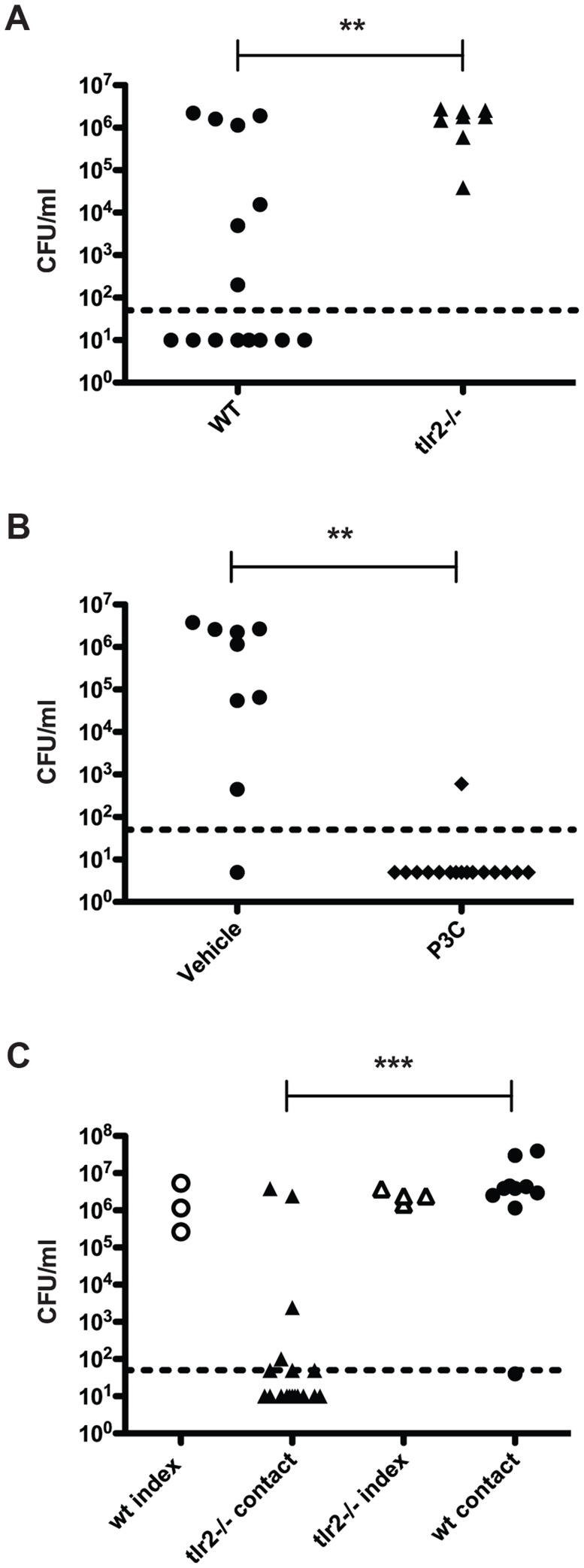
TLR2 stimulation limits pneumococcal transmission. **A.** *tlr2^−/−^* mice were infected as described in Figure 1A, and bacterial loads in nasal lavage fluid of contact mice are shown (filled triangles). Data from wildtype contacts (replicated from figure 1B) are also shown for comparison (filled circles). **B.** Wildtype mice were infected as described in Figure 1A, and all pups were given Pam3Cys or PBS (vehicle control) intranasally on days 8, 10, and 12. Mice were sacrificed on day 14, and bacterial loads in lavage fluid of contact mice are shown. **C.** Age-matched wildtype and *tlr2^−/−^* pups were split into index and contact groups and cross-fostered such that either the index were wildtype and the contacts were *tlr2^−/−^* or that the index were *tlr2^−/−^* and the contacts were wildtype. The infection scheme described in Figure 1A was then followed, and bacterial loads in nasal lavage fluid are shown, with open symbols denoting index mice and filled symbols representing contact mice. ** indicates *p*<0.01 by Mann-Whitney test.

We then repeated the transmission experiment using *tlr2^−/−^* mice. All *tlr2^−/−^* contact mice acquired pneumococcal colonization and were colonized at high levels (Figure 2A). As TLR2 deficiency led to increased transmission, we reasoned that stimulation of TLR2 could limit bacterial spread. To test this, we performed a transmission experiment with wildtype mice and intranasally administered the TLR2 agonist Pam3Cys three times over the course of the exposure period (days 8, 10, and 12). As depicted in Figure 2B, transmission was significantly less efficient in treated animals than in control litters, with only one mouse out of sixteen acquiring colonization.

We hypothesized that the increase in transmission efficiency seen in *tlr2^−/−^* animals could be due to either increased spread by the index mice or increased susceptibility in the contact mice. To address this question, we assessed whether the transmission phenotype was dependent on the index or contact mice in a mixed litter experiment. Age-matched litters of wildtype and *tlr2^−/−^* mice were inoculated with *S. pneumoniae* as described above, but the index mice from each litter were switched, such that *tlr2^−/−^* index were co-housed with wildtype contacts and wildtype index were co-housed with *tlr2^−/−^* contacts. After the six-day exposure period, only 39% of the *tlr2^−/−^* contacts housed with wildtype index mice had detectable levels of colonization (Figure 2C). This was not significantly different from the wildtype contact group in the previous experiments, but was significantly different from the *tlr2^−/−^* contact group (*p* = 0.0014). On the contrary, 89% of wildtype contacts housed with *tlr2^−/−^* index mice became infected (Figure 2C). This was not significantly different from the *tlr2^−/−^* contact group, but was significantly different from the wildtype contact group (*p* = 0.0013). These results indicate that the increased transmission phenotype seen in the *tlr2^−/−^* mice is linked to the index mouse, which we postulated was due to increased spread of the bacterium from these mice. However, this effect was not due to an increased bacterial load in the *tlr2^−/−^* mice compared to the wildtype. We thus concluded that TLR2 deficiency likely did not cause an increase in susceptibility to bacterial acquisition in this model.

### Influenza infection results in neutrophil influx to the nasopharynx

**Figure 3:**
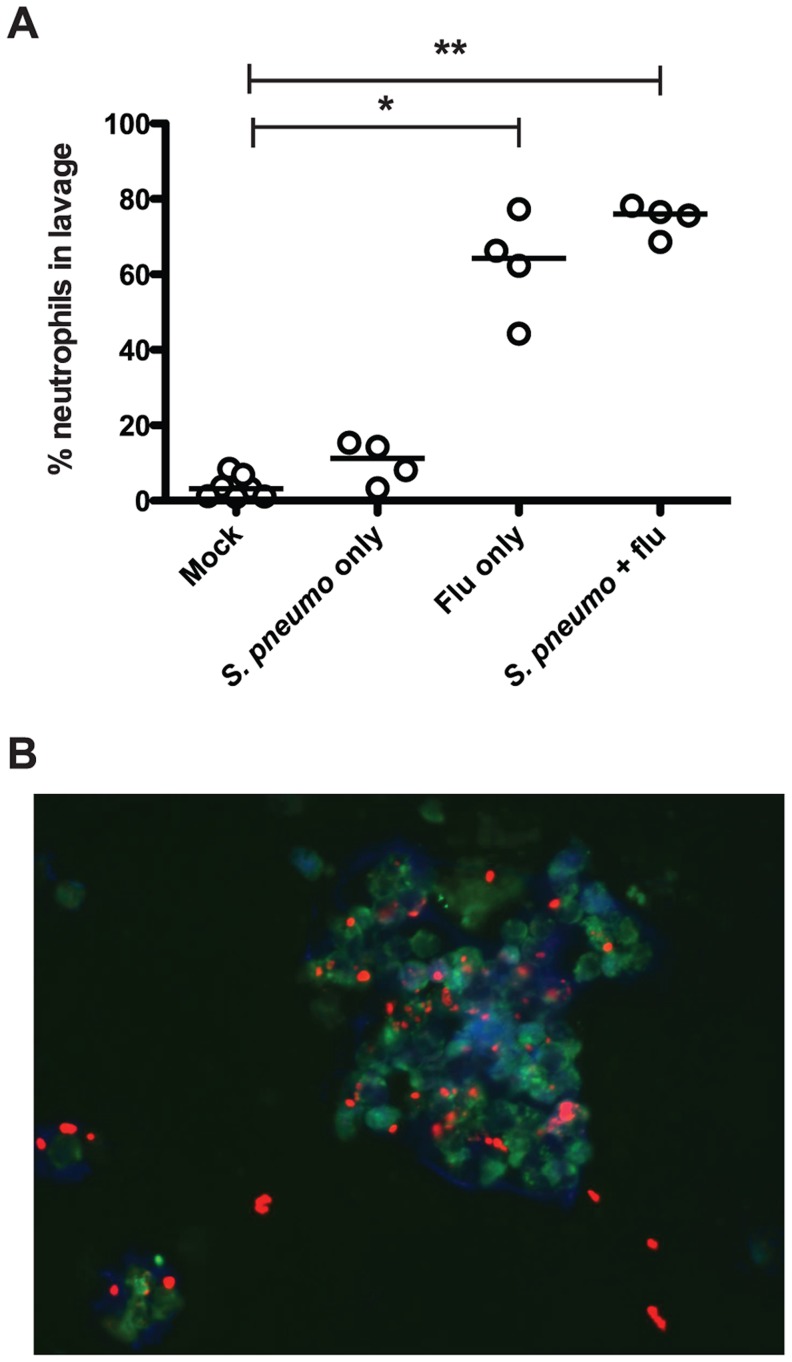
Influenza infection results in neutrophil influx to the nasopharynx. Wildtype mice were infected as index mice with the listed agents. On day 14, mice were euthanized, and nasal lavage was performed with PBS. **A.** A 100 µl sample of each lavage was analyzed by flow cytometry to detect total numbers of neutrophils (Ly6G^+^, CD11b^+^). Each dot represents the percentage of total events that were neutrophils per mouse. Statistical significance was assessed using the Kruskal-Wallis test followed by Dunn's post-test, and * indicates *p*<0.05, while ** denotes *p*<0.01. **B.** Immunofluorescence image (representative). Ten microliter samples of nasal lavage fluid were spotted onto a glass slide, fixed, and stained with DAPI (blue) and antibodies against Ly6B (green) and type 23F pneumococcal capsule polysaccharide (red).

We next assessed the innate immune response to infection with influenza and pneumococcus, both separately and in the context of co-infection, in our infant mouse model. To test this, we infected wildtype mice as index pups according to the schematic in Figure 1A, giving PBS doses when appropriate for single or mock-infected groups. Nasal lavage was performed on day 14, and a sample of lavage fluid was stained with antibodies against a panel of immune cells (Ly6G, CD11b, F/480 and CD4). While no significant influx of macrophages or T cells was seen (data not shown), we noticed significant differences in neutrophil populations, as seen in Figure 3A. Neutrophils (Ly6G^+^ and CD11b^+^ events) comprised very little of the total cell count for both mock-infected mice and those given *S. pneumoniae* alone. However, when influenza was present, both in single infection and in co-infection with the pneumococcus, we observed a significant neutrophil influx, comprising 62.6% and 74.7% of the total cell infiltrate, respectively (Figure 3A).

We also visualized neutrophils and bacteria in the lavage fluid via immunofluorescence microscopy. The representative image in Figure 3B shows multiple intact pneumococci associated with a cluster of neutrophils. These clusters of neutrophils were not seen in mice that were mock infected or infected with the pneumococcus alone (data not shown). Taking these results together, we posit that influenza infection sparks neutrophil influx into the nasopharynx, and that this acute inflammatory response is ineffective at clearing the bacteria, but facilitates the spread of bacteria amongst littermates.

### Mice deficient in TLR2 display increased inflammation

**Figure 4:**
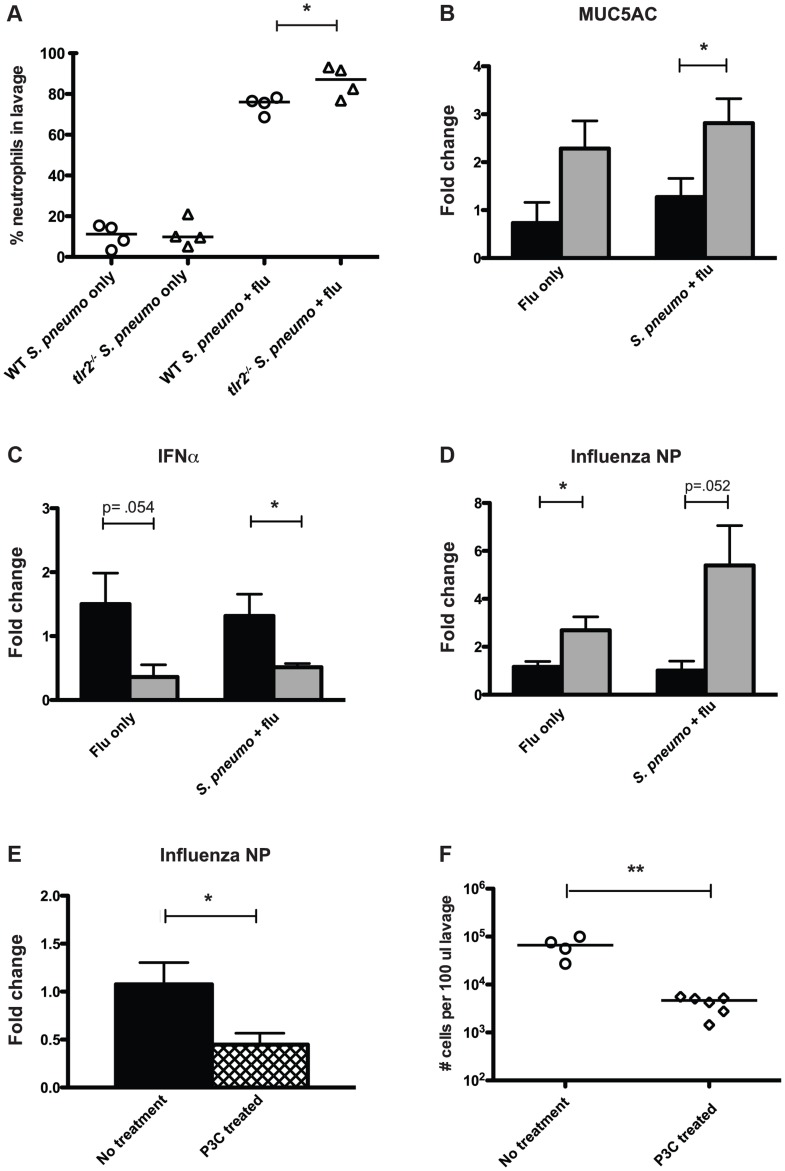
Mice deficient in TLR2 display increased inflammation and increased viral titers following influenza infection. **A.** Mice of listed genotypes were infected with specified agents, as above, and nasal lavage fluid was stained and analyzed by flow cytometry. Percentage of total events staining positive for neutrophils are shown, with each dot representing one mouse. Asterisk denotes *p*<0.05 using an unpaired Student's *t* test. **B–D.** Wildtype (black bars) and *tlr2^−/−^* (gray bars) mice were infected as index mice with either *S. pneumoniae* and influenza or influenza alone as described in Figure 1A. Mice were sacrificed on day 14 (day 11 for panel C), and following nasal lavage with 200 µl PBS, a second lavage with 600 µl RLT lysis buffer was performed, from which RNA was then extracted. Quantitative real-time PCR was performed to determine RNA levels of MUC5AC (B), IFNα (C), and influenza nucleoprotein (D). **E.** Wildtype mice were infected as index mice with *S. pneumoniae* and influenza. For P3C treated mice (hatched bar), 10 µg of Pam3Cys was administered intranasally on days 8, 10, and 12. RNA was extracted from nasal lavages as described above, and levels of influenza nucleoprotein were assessed by qRT-PCR. For B–E, fold change in *tlr2^−/−^* and P3C-treated mice relative to wildtype was determined using the ΔΔCT method with GAPDH as an endogenous control. Bars indicate mean of 6 mice per condition (±SEM), and asterisks denote statistically significant difference using Student's *t* test (*p*<0.05). **F.** Wildtype mice were infected as index animals, and were given Pam3Cys intranasally on days 8, 10, and 12, or left untreated. Flow cytometry analysis was performed on stained nasal lavages, and total cells counts in 100 µl lavage fluid were determined. Each dot represents one mouse, and statistical significance was determined using a Mann-Whitney test, with ** denoting *p*<0.01.

Based on the observation that TLR2 deficiency led to increased transmission, we hypothesized that the innate immune response to co-infection with the pneumococcus and influenza differed between these two groups. When we analyzed the neutrophil content of lavage fluid from co-infected *tlr2^−/−^* mice, we found neutrophils to make up a significantly higher percentage of the total cells than in wildtype mice (mean 86.1%, Figure 4A). This was not due to a difference in response to the pneumococcus, as these percentages were not different between wildtype and *tlr2^−/−^* infected with *S. pneumoniae* alone (Figure 4A). As this result suggested that there was more inflammation in co-infected *tlr2^−/−^* mice, we also compared mucus production by analyzing relative expression levels of *Muc5ac*, the primary secreted mucin of the nasopharynx, by qRT-PCR [24]. We found that in co-infected *tlr2^−/−^* mice, *Muc5ac* transcript levels were on average 2.8-fold higher than in co-infected wildtype mice (Figure 4B).

Recent work has shown that TLR2-dependent signaling can induce anti-viral responses in the form of type I IFN production [22], [23]. Considering these findings, we hypothesized that *tlr2^−/−^* mice display a weakened anti-viral response, rendering them more susceptible to influenza infection leading to heightened inflammation. To assess the anti-viral responses produced by the mice used in our model, we examined IFNα expression levels by qRT-PCR at an early time point after influenza inoculation (3 days) and found that co-infected *tlr2^−/−^* mice showed a 2.6-fold reduction in IFNα transcript compared to wildtype (Figure 4C). Additionally, levels of viral RNA were 5.4-fold higher in *tlr2^−/−^* mice than wildtype, suggesting that the increased inflammation observed could be due to increased viral titers in the *tlr2^−/−^* host (Figure 4D). This increase in viral levels was not dependent on the presence of pneumococcus, as evidenced by the ∼3-fold increase in viral RNA in *tlr2^−/−^* mice infected with influenza alone. Thus, increased inflammation appears to be associated with increased transmission of *S. pneumoniae* by infected hosts. Supporting this, mice treated with the TLR2 agonist Pam3Cys displayed both lower viral titers (Figure 4E) and subsequently, less of an inflammatory response, with fewer total cells in the nasopharyngeal infiltrate (Figure 4F).

### Influenza infection induces bacterial shedding at a level high enough to infect infant mice

**Figure 5:**
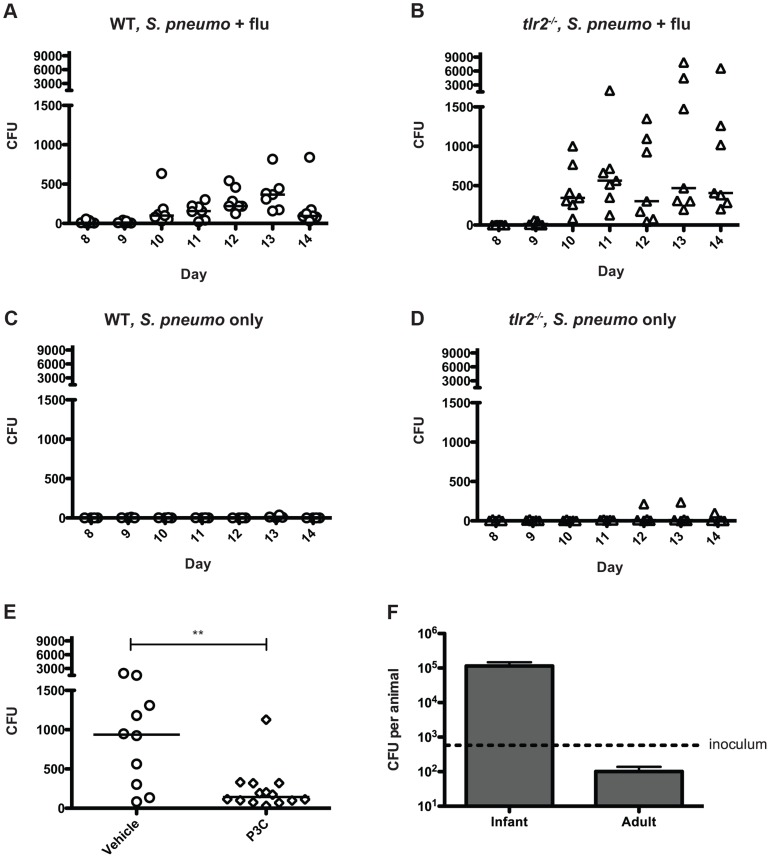
Influenza infection induces bacterial shedding at a level high enough to infect infant mice. **A–E.** Wildtype (A, C, and E) and *tlr2^−/−^* mice (B and D) were infected with either *S. pneumoniae* and influenza (A, B and E) or *S. pneumoniae* alone (C and D) as described in Figure 1A. Each day (starting on day 8), the nose of each mouse was gently pressed onto an agar plate 10 times, exhaled bacteria were spread, and plates were incubated overnight. Each symbol represents the bacterial count from one animal on each day. In panel E, Pam3Cys or PBS alone (vehicle control) was administered on days 8, 10 and 12. Colony counts from days 10 and 11 are combined for comparison. **F.** Infant (7 day old) and adult (6 week old) mice were inoculated with ∼500 (dotted line) CFU of strain P1121 in PBS. One day post inoculation, the mice were sacrificed, nasal lavage was performed and lavage fluid was serially diluted and plated on selective media to enumerate bacteria.

Taken together, our data have suggested that inflammation promotes bacterial spread, and thus we hypothesized that increases in inflammation and mucus production lead to increased bacterial shedding in the form of nasal secretions. In order to determine the number of bacteria being shed in this manner, we adapted a method in which the nose of the mouse is gently pressed onto a nutrient agar plate and exhaled bacteria are then quantified [25]. We observed that in both wildtype and *tlr2^−/−^* co-infected mice, detectable levels of bacteria were shed throughout the experiment, starting on day 10 (Figure 5A,B). When bacterial counts from days 10–14 are compared between groups, the *tlr2^−/−^* mice shed significantly more bacteria then the wildtype group over the course of the exposure window (*p*<0.0001). As TLR2 deficiency led to increased shedding, we reasoned that stimulation of this receptor could limit shedding, and thus we repeated the wildtype shedding experiment, including three intranasal administrations of Pam3Cys over the exposure period. The treated animals shed significantly fewer bacteria than the control litter (Figure 5E), demonstrating that TLR2 stimulation can limit shedding. Bacterial shedding was dependent on influenza co-infection for both groups, as mice infected with *S. pneumoniae* alone did not shed appreciable amounts of bacteria at any point in the experiment (Figure 5C,D).

To determine if the numbers of bacteria shed were sufficient to infect contact mice, we intranasally administered 500 CFU of *S. pneumoniae* (∼ID_50_ for adult mice) to 7-day-old pups and assessed colonization levels the following day. We found that this was a sufficient dose to establish colonization with a robust increase in bacterial density over the inoculum size in the infant mouse nasopharynx, with 100% of pups colonized. Most adult mice, in contrast, were not consistently colonized by this low dose and those that became colonized had bacterial numbers below that of the inoculum – a result that correlated with the lack of pneumococcal transmission observed among adult mice (Figure 5F). In co-infected infant pups, from days 10–14, approximately 14% of wildtype mice regularly shed >500 colonies, while 43% of *tlr2^−/−^* mice consistently shed at this level or higher, corresponding to the rates of transmission in these two groups. In contrast, none of the mice given PBS instead of influenza shed above this level. These experiments suggest that TLR2-dependent inflammation induced by influenza infection promotes shedding of *S. pneumoniae* through nasal secretions, and the contact between infected and uninfected infant mice is sufficient to mediate bacterial transmission from host to host.

## Discussion

This study aimed to expand existing knowledge of the transmission of respiratory bacterial pathogens by specifically analyzing spread of *Streptococcus pneumoniae* in an infant mouse model. Transmission of bacterial pathogens is critical to their success but has long been a black box in the study of pathogenesis due to a lack of tractable animal models. An infant mouse model utilizing influenza co-infection has been recently introduced [14], [15]. These studies have established a preliminary link between inflammation induced by infection and spread of the bacterium. Here, we sought to identify specific host factors that contribute to this process and to gain further insight into the mechanisms responsible for transmission in this model.

Our results demonstrate a role for the innate immune receptor TLR2 in transmission of the pneumococcus in a flu-dependent manner. The findings in our report are consistent with previous studies in adult mice, which suggested that TLR2 stimulation by either commensal bacteria [26] or a synthetic agonist [21] is protective against flu infection. Previous in vitro studies have also demonstrated a role for TLR2-mediated signaling in induction of type I IFNs [22], [23]. Our work adds in vivo data to this model, demonstrating that expression of IFN α, a key component of the anti-viral response, was diminished in *tlr2^−/−^* mice compared to wildtype mice. These findings solidify a link between TLR2 activity and type 1 IFN expression.

Importantly, influenza co-infection was required for transmission to occur; this was true for both wildtype and *tlr2^−/−^* experimental groups. Both infection with influenza alone and co-infection resulted in a significant inflammatory influx to the nasopharynx, with the largest proportion of the cellular infiltrate comprised of neutrophils. In *tlr2^−/−^* mice, this response was even more pronounced, with a significantly higher percentage of neutrophils present than in the wildtype samples, while we did not observe any differences in macrophages or other cell types recruited. The host response to increased viral titers also correlated with higher expression of the mucin Muc5ac. We hypothesize that these higher levels of virus stimulate an exaggerated acute inflammatory response that drives an increase in nasal secretions, consisting of mucus and inflammatory cells, providing an exit vehicle for the pneumococcus. As the presence of pneumococcus adds relatively little to inflammation, our findings suggest that it is primarily the host response to the virus that provides this vehicle for shedding, with the bacterium acting as a passenger.

The pneumococcus can thus take advantage of the heightened inflammatory state present in co-infected index animals. The observation that there is very little inflammation seen in the context of a pneumococcal single infection explains why transmission is not detected when the animals are not given influenza. The few neutrophils that are recruited to the airway lumen in response to pneumococcal colonization have a limited ability to take up the organism in the absence of specific antibody, and depletion of neutrophils has no effect on bacterial clearance in adult singly infected mice [27]. Additionally, the neutrophil influx observed in influenza-infected lavages did not appear to be effective at lysing the bacterium, as indicated by the predominance of intact bacterial cells in immunofluorescence images. Thus, the secretions resulting from robust inflammation are required for transmission, but this inflammatory response must also be ineffective at killing the bacterium.

We also demonstrate here that the inflammation induced by influenza infection promotes bacterial shedding from index mice at or above a level sufficient to infect uninoculated contact mice. Transmission could be a consequence of bacterial shedding above this threshold level. We conclude that the increased inflammation in *tlr2^−/−^* mice is due mostly to diminished sensing of the virus and inability to control viral infection. Another consideration is that while *tlr2−/−* genotype itself does not bias the composition of the host microbiota [28], it likely causes differences in sensing of the flora. Although previous studies have shown that the microbiota can affect the immune response to influenza, the contribution of the colonizing pneumococci to the inflammatory response was small in comparison to influenza. This was the case even though pneumococcal colonization itself stimulates TLR2 signaling [19]. Thus, it appears that the increased viral load observed in *tlr2^−/−^* mice was sufficient to lead to a heightened inflammatory response through sensing by other viral PRRs resulting in more copious purulent mucus secretions. These secretions carry live bacteria, which are then shed in increased numbers. As the nursing mother piles infant mice in close proximity to one another, there are thus ample opportunities for the bacteria to spread from one host to another. These studies also help to explain the transmission differences between adult and infant mice, namely that transmission has not been observed in similarly treated adults because of the higher inoculum required to establish robust colonization in adults.

This is the first study to implicate a specific host factor in transmission of the bacterial pathogen *S. pneumoniae*. While stimulation of TLR2 limits transmission, approximately half of wildtype contact mice acquire the bacterium, indicating that other components of the innate immune system must contribute to the inflammation and shedding necessary for this process. For instance, stimulation of other pattern recognition receptors that respond to influenza, such as TLR3, TLR7, and RIG-I, could affect pneumococcal transmission, as shown for TLR2. Signaling downstream of other viral PRRs has yet to be fully explored in the context of transmission. The model detailed here thus shows much promise for investigating these additional microbial and host factors to determine the complete mechanism behind bacterial shedding and its consequences for host-to-host transmission.

## Materials and Methods

### Ethics statement

This study was conducted according to the guidelines outlined by National Science Foundation Animal Welfare Requirements and the Public Health Service Policy on the Humane Care and Use of Laboratory Animals. The protocol was approved by the Institutional Animal Care and Use Committee, University of Pennsylvania Animal Welfare Assurance Number A3079-01, protocol number 803231.

### Bacterial and viral strains and culture conditions

*S. pneumoniae* strains were grown statically in tryptic soy broth (BD, Franklin Lakes, NJ) at 37°C in a water bath. All studies described here utilized strain P1121, a serotype 23F isolate that has been previously used for human carriage studies [29]. Bacteria were stored in 20% glycerol at −80°C. Influenza A/HKx31 (H3N2) was grown in the allantoic fluid of 10-day embryonated chicken eggs (B&E Eggs) and stored at −80°C. Viral concentrations for infection were determined by titration in Madin-Darby Canine Kidney cells, as described previously [30].

### Mice

All experiments using animals were approved by the Institutional Animal Care and Use Committee of the University of Pennsylvania, and mice were housed in accordance with IACUC protocols. Wildtype C57BL/6 mice were originally obtained from The Jackson Laboratory (Bar Harbor, ME). The *tlr2^−/−^* mice have been described previously [19]. All mice were bred and maintained in a conventional animal facility, and both male and female pups were used.

### Infant mouse transmission experiments

Mice were bred under specific pathogen-free conditions at the University of Pennsylvania. Four days after pups were born, 1–2 index mice (such that ratio of index∶contact was always 1∶3 to 1∶4) were randomly selected from each litter and inoculated with 2000 CFU of *S. pneumoniae* suspended in 3 µl PBS intranasally. Inoculation was performed atraumatically with a blunt pipette tip without anesthesia, and index pups were returned to the litter. When pups were 8 days old, all infants were given 2×10^2^–2×10^4^ TCID_50_ Influenza A/HKx31 in 3 µl PBS intranasally. This H3N2 isolate was chosen because it replicates well in the mouse upper respiratory tract without causing disease [30]. For mock infections, sterile PBS was given. When indicated, pups were treated with the TLR2 agonist Pam3Cys on days 8, 10, and 12. Pam3Cys (Invivogen) was resuspended to a concentration of 2 mg/ml in sterile water, and 10 µg doses were given intranasally. On day 14, all pups were euthanized by CO_2_ asphyxiation. To quantify bacteria, the trachea was exposed, cannulated, and flushed with 200 µl sterile PBS. PBS lavages were serially diluted and plated on tryptic soy agar containing neomycin (20 µg/ml) to minimize the growth of contaminants. To obtain RNA from the epithelium, a second lavage was performed with 600 µl of RLT lysis buffer (QIAGEN) and stored at −80°C until needed.

### Flow cytometry

Nasal lavage samples (100 µl per mouse) were stained with the following fluorescent antibodies: CD4-FITC, Ly6G-PE, CD11b-perCP and F4/80-APC (eBioscience) after blocking with FC Block at 4°C. Cells were then fixed with 1% paraformaldehyde and assayed using a BD FACSCalibur the following day. Data were gathered using CellQuest Pro software (BD), analyzed using FlowJo software (TreeStar), and graphed with Prism 5 (GraphPad).

### Immunofluorescence

Undiluted nasal lavage fluid was spotted onto glass microscope slides and allowed to air-dry, and then fixed and stained essentially as described [19], using rabbit serum against type 23F pneumococcus (1∶5000) and rat anti-mouse α-Ly6B (1∶100, AbD serotec) primary antibodies with anti-rabbit-Cy3 and anti-rat-FITC conjugated secondary antibodies (both 1∶600), respectively, along with DAPI staining. Immunofluorescence images were collected using a Nikon Eclipse E600 (Nikon Instruments Inc.) equipped with a liquid crystal (Micro*Color RGB-MS-C; CRi Inc.) and a charge-coupled device digital camera.

### RNA extraction and qRT-PCR analysis

RNA was isolated from the epithelium lining the mouse nasopharynx following lavage with 600 µl RLT lysis Buffer using an RNeasy Mini Kit (QIAGEN) according to the manufacturer's instructions. For all experiments except IFNα analysis, lavages were collected at day 14. IFNα levels were measured in lavage collected on day 11. cDNA was generated from each sample using a high-capacity reverse transcription kit (Applied Biosystems). Approximately 10 ng cDNA was used as a template in reactions with forward and reverse primers (0.5 µM) and SYBR Green (Applied Biosystems), according to the manufacturer's instructions. Reactions were carried out using the StepOnePlus Real-Time PCR system, and fold changes were calculated using the ΔΔCT method (Applied Biosystems). GAPDH was used as an endogenous control. The following primers were used in reactions: influenza nucleoprotein – F 5′-CAGCCTAATCAGACCAAATG-3′, R 5′-TACCTGCTTCTCAGTTCAAG-3′; MUC5AC – F 5′-CCATGCAGAGTCCTCAGAACAA-3′, R 5′-TTACTGGAAAGGCCCAAGCA-3′; GAPDH – F 5′-TGTGTCCGTCGTGGATCTGA-3′, R 5′-CCTGCTTCACCACCTTCTTGAT-3′, IFNα – F 5′-TCTGATGCAGCAGGTGGG-3′, R 5′-AGGGCTCTCCAGACTTCTGCTCTG-3′.

### Bacterial shedding assay

Infant mice were infected as “index mice” as described above for the transmission model. From day 8 to day 14, daily sampling was performed for each mouse, in which the nose of the mouse was gently pressed onto tryptic soy agar containing neomycin (20 µg/ml) 10 times to obtain a representative sample. The mouse was then returned to the cage, and exhaled bacteria were spread across the surface of the plate with a polyester-tipped swab. Plates were grown overnight at 37°C with 5% CO_2_ and colonies were enumerated the following day.

### Mouse colonization

Six to eight-week-old (adult) and seven-day-old (infant) unanesthetized wildtype C57BL/6 mice were intranasally inoculated with 500 CFU of *S. pneumoniae* P1121. Twenty-four hours later, mice were sacrificed by CO_2_ asphyxiation, and tracheas were exposed and cannulated, then flushed with 200 µl PBS. Lavage fluid was collected from the nares, serially diluted, and plated on tryptic soy agar. Colonies were enumerated after overnight incubation at 37°C with 5% CO_2_.

## Supporting Information

Figure S1 **Timing of pneumococcal acquisition does not affect transmission to littermates.** Index (inoculated contact) pups were inoculated with *S. pneumoniae* on day 9 instead of day 4, but otherwise treated as detailed in Figure 1A. For all panels, dotted line indicates limit of detection (50 CFU).
(EPS)
